# Supplementary material for: Biotic homogenization, lower soil fungal diversity and fewer rare taxa in arable soils across Europe
Source: Nat Commun. 2024 Jan 6;15:327. doi: 10.1038/s41467-023-44073-6 (PMC10771452; doi:10.1038/s41467-023-44073-6)
Supplement: Supplementary file 1 — Supplementary Information [file 41467_2023_44073_MOESM1_ESM.pdf]

Supplementary Methods

Amplicon library preparation

Fungal ITS region was amplified using the PacBio SMRT Sequel platform with the primers ITS1F (CTTGGTCATTTAGAGGAAGTAA) and ITS4 (TCCTCCGCTTATTGATATGC) targeting the entire ITS region [1]. Each primer was tagged with a 5-nucleotide-long padding sequence and an 8-nucleotide-long barcode. We conducted a two-step PCR on a Biorad PCR Instrument (Biorad, Hamburg, Germany) using the 5PRIME HotMaster Taq DNA Polymerase (Quantabio, Beverly, MA, USA) in 20 µl of reaction mixture. First PCR reaction were performed with two technical replicates for each DNA sample. Thermal cycling conditions comprised a 2 min of initial denaturation at 94°C followed by 25 cycles of 45s denaturation at 94°C, 1 min annealing at 55°C and 1 min elongation at 72°C, and a final elongation of 10 min at 72°C. Products of two replicates were then pooled and cleaned of remaining primers and dNTPs using Antarctic phosphatase. The second amplification had only 10 cycles, otherwise the thermal cycling conditions were the same. The number of cycles was kept low to minimize chimera formation and to be able to deduce sequence abundance in a semi-quantitative manner [2]. Amplicons were loaded on a 1% agarose gel to examine PCR efficiency and the lack of PCR amplicons in non-template control reactions. After PCR, replicates were pooled for each sample and the concentration of amplicon DNA was determined using PicoGreen (Molecular Probes Inc., Eugene, Oregon, USA) on the Varian Turbo GTI fluorescence plate reader (Varian Inc., Poalo, CA, USA). Three amplicon libraries (~2 µg each) were assembled by combining barcoded DNA samples and purifying twice with the Agencourt AMPure XP PCR Purification system (Beckman Coulter, IN, USA). For each library, a final volume of 100 µl was obtained by eluting in sterile miliQ water. Later, the hairpin sequencing adapters (SMRTbellTM templates, DNA Template Prep Kit 2.0 (250bp-<3Kb)) were ligated to the purified amplicon libraries following the blunt-end ligation protocol of PacBio (2kb library preparation; Pacific Biosciences, CA) at the Functional Genomic Centre Zurich (FGCZ, Zurich, Switzerland; http://www.fgcz.ch). Sequencing libraries

were purified with AMPure and examined for quality (Bioanalyzer, Agilent, CA, USA) and quantity (Qubit 1.0, Thermo Fischer Scientific, NY, USA). Libraries were finalized for sequencing by annealing the sequencing primers to the SMRTbells and binding of the DNA polymerase to the template complex. The sequencing libraries were prepared using P6/C4 chemistry (DNA/Polymerase Binding Kit P6, DNA Sequencing Reagent 4.0) on the PacBio® Sequel Instrument. The PacBio SMRT Portal (https://www.pacb.com/products-and-services/analytical-software/smrt-analysis/) was employed to process the raw sequences and extract the circular consensus sequences of at least five passes yielding in similar error rates as 454 or MiSeq sequencing platforms [3].

Fastq files obtained from the PacBio runs were quality filtered using the PRINSEQ-lite v0.20.4 [4]. Filtering parameters were: GC range 30-70, minimum mean quality score of 20, no ambiguous nucleotides, low sequence complexity filter with a threshold of 30 in the DUST algorithm. In a next step, the reads were demultiplexed using an in-silico PCR approach as part of USEARCH v11 [5] allowing max 1 mismatch in the barcode-primer sequence but not at the 3-prime ends. The amplicon size range was set to 100 – 2000 but all amplicons with additional primer sites (concatenated amplicons - multi-primer artefacts) were removed. Sequences were then clustered into operational taxonomic units (OTUs), based on 97% similarity, using the UPARSE pipeline (Edgar 2013). Taxonomical information was predicted to OTUs based on the UNITE database (V7.2) [6] using SINTAX. The taxonomic prediction were verified using ITSx [7] and non-fungal OTUs were excluded. Singletons (OTUs that only occur once) and OTUs with low abundance (relative abundance less than 0.5% in total and less than 0.5% in each sample) were excluded. A total of 17 phyla, 42 classes, 90 orders, 187 families and 293 genera were classified. Only 6% OTUs were identified at the species level. Nearly half (49.4%) of the OTUs were classified to at least the class level, accounting for 80.2% of total abundance.

Acquisition of climatic data

Climatic data at a resolution of 10 minutes (around 18.5 km at latitude 40 degrees) including 19 bioclimatic variables were obtained from the WorldClim database (http://www.worldclim.org), including 11 variables derived from monthly temperature values and 8 variables derived from monthly precipitation values [8]. This database summarizes monthly climate conditions, and has been previously used in biogeographical studies [9]. We extracted the following data for each site (averages for the years 1970-2000): annual mean temperature; mean diurnal range (mean of monthly (maximum temperature - minimum temperature)); isothermality (mean diurnal Range / temperature annual range) (* 100); temperature seasonality (standard deviation *100); maximum temperature of warmest month; minimum temperature of coldest month; temperature annual range (maximum temperature of warmest month - minimum temperature of coldest month); mean temperature of wettest quarter; mean temperature of driest quarter; mean temperature of warmest quarter; mean temperature of coldest quarter; annual precipitation; precipitation of wettest month; precipitation of driest month; precipitation seasonality (coefficient of variation); precipitation of wettest quarter, precipitation of driest quarter; precipitation of warmest quarter; and precipitation of coldest quarter.

.

Table S1. Percentages of shared and unique OTUs in arable lands and grassland of five countries. Sample sizes were standardized to ensure equal number of arable and grassland fields for each country.

Country	% of OTUs only in arable land	% of OTUs only in grassland	% of OTUs shared by both land use types
Spain	20.60%	40.60%	38.80%
France	26.50%	40.70%	32.90%
Switzerland	22.30%	36.20%	41.50%
Germany	18.70%	44.50%	36.80%
Sweden	27.30%	43.10%	29.60%

Table S2. Members of major fungal classes that changed significantly across two land-use types. Welch's t-test was performed to assess whether abundances differed significantly between arable lands and grasslands. Statistical significance: * p value < 0.05; ** p value <0.01; *** p value <0.001; n.s. not significant.

**Sordariomycetes**	**Pezizomycetes**	**Mortierellomycetes**	**Tremellomycetes**	**Dothideomycetes**	**Agaricomycetes**	**Leotiomycetes**	**Eurotiomycetes**	**Pezizomycetes**
o__Sordariales ***	f__Pyronemataceae ***	f__Mortierellaceae ***	o__Cystofilobasidiales **	o__Pleosporales ***	o__Cantharellales ***	o__Helotiales ***	o__Chaetothyriales ***	f__Pyronemataceae ***
o__Hypocreales ***	f__Pezizaceae n.s.		o__Tremellales ***	o__Capnodiales ***	o__Agaricales n.s.	o__Erysiphales *	o__Eurotiales ***	f__Pezizaceae n.s.
o__Glomerellales ***	f__Ascobolaceae **		o__Trichosporonales ***	o__Dothideomycetes_ord_Incertae_sedis **	o__Sebacinales ***	o__Leotiomycetes_ord_Incertae_sedis ***	o__Onygenales n.s.	f__Ascobolaceae **
o__Microascales ***	f__Tuberaceae *		o__Filobasidiales ***	o__Venturiales *	o__Thelephorales n.s.		o__Verrucariales **	f__Tuberaceae *
o__Branch06 ***	f__Sarcoscyphaceae n.s.			o__Tubeufiales *	o__Auriculariales ***		o__GS37 ***	f__Sarcoscyphaceae n.s.
o__Xylariales ***				o__Dothideales ***	o__Corticiales n.s.			
o__Ophiostomatales *					o__Trechisporales n.s.			
o__Diaporthales *					o__Polyporales ***			
o__Chaetosphaeriales ***					o__Atheliales n.s.			
o__Myrmecridiales n.s.					o__Boletales n.s.			
o__Trichosphaeriales *					o__Russulales ***			
o__Magnaporthales *					o__Geastrales n.s.			
o__Coniochaetales n.s.					o__Hymenochaetales n.s.			

Table S3. Members of major fungal classes that changed significantly across five European countries. Welch's t-test was performed to assess whether abundances differed significantly between arable lands and grasslands. Statistical significance: * p value < 0.05; ** p value <0.01; *** p value <0.001; n.s. not significant.

**Sordariomycetes**	**Pezizomycetes**	**Mortierellomycetes**	**Tremellomycetes**	**Dothideomycetes**	**Agaricomycetes**	**Leotiomycetes**	**Eurotiomycetes**
o__Sordariales ***	f__Pyronemataceae n.s.	f__Mortierellaceae *	o__Cystofilobasidiales **	o__Pleosporales	o__Cantharellales n.s.	o__Helotiales **	o__Chaetothyriales n.s.
o__Hypocreales n.s.	f__Pezizaceae n.s.		o__Tremellales n.s.	o__Capnodiales **	o__Agaricales ***	o__Erysiphales n.s.	o__Eurotiales ***
o__Glomerellales n.s.	f__Ascobolaceae n.s.		o__Trichosporonales *	o__Dothideomycetes_ord_Incertae_sedis ***	o__Sebacinales ***	o__Leotiomycetes_ord_Incertae_sedis **	o__Onygenales ***
o__Microascales n.s.	f__Tuberaceae *		o__Filobasidiales n.s.	o__Venturiales n.s.	o__Thelephorales **		o__Verrucariales n.s.
o__Branch06 ***	f__Sarcoscyphaceae n.s.			o__Tubeufiales *	o__Auriculariales *		o__GS37 n.s.
o__Xylariales n.s.				o__Dothideales **	o__Corticiales n.s.		
o__Ophiostomatales n.s.					o__Trechisporales n.s.		
o__Diaporthales n.s.					o__Polyporales n.s.		
o__Chaetosphaeriales ***					o__Atheliales n.s.		
o__Myrmecridiales n.s.					o__Boletales n.s.		
o__Trichosphaeriales n.s.					o__Russulales **		
o__Magnaporthales n.s.					o__Geastrales n.s.		
o__Coniochaetales **					o__Hymenochaetales n.s.		

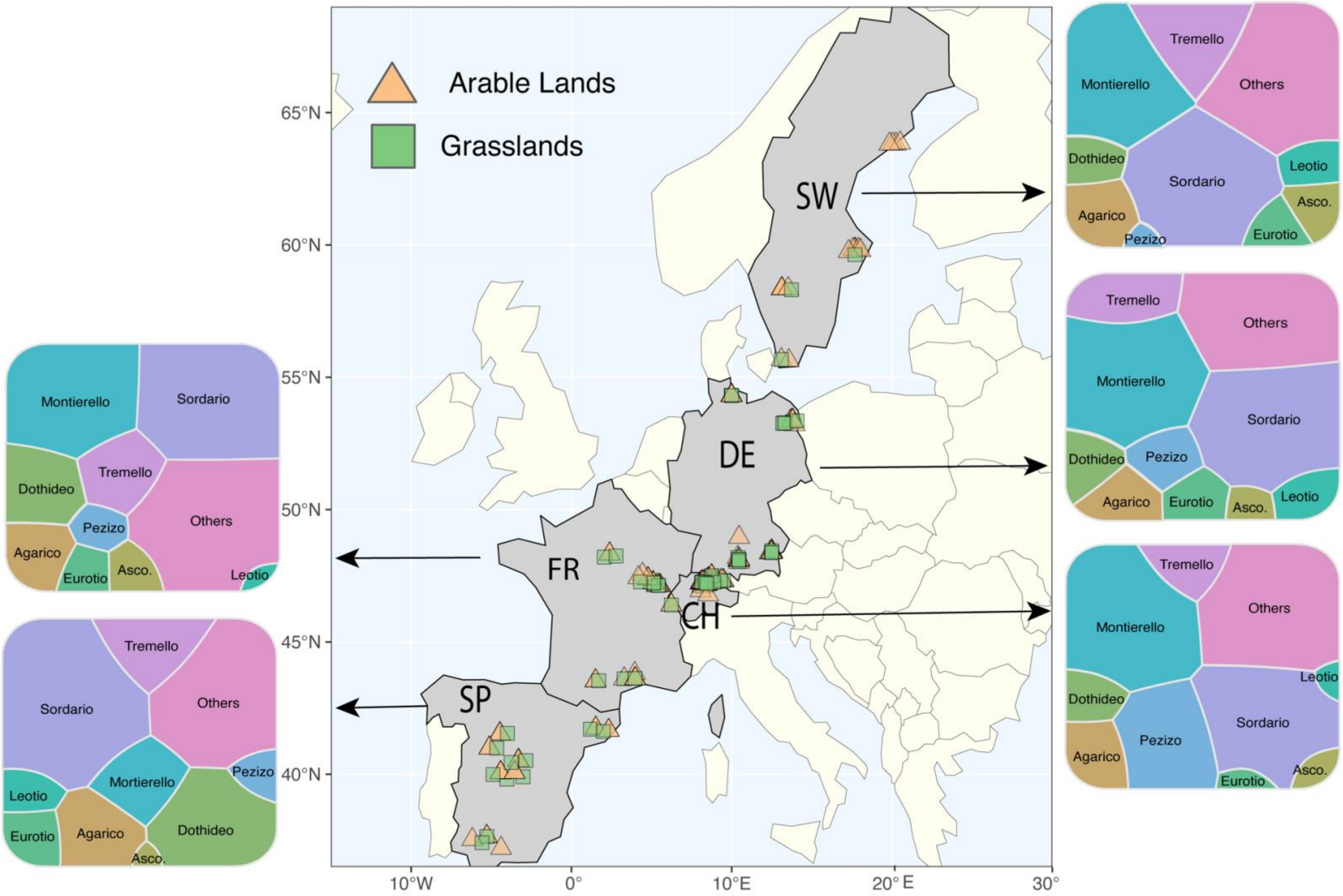

Figure S1. Field sites and the overall fungal composition across a 3,000-km gradient in Europe. Voronoi diagrams showing the proportional abundance of top ten fungal classes in Sweden (SW), Germany (DE), Switzerland (CH), France (FR), and Spain (SP). The major classes groups are: Mortierellomycetes, Sordariomycetes, Dothideomycetes, Tremellomycetes, Agaricomycetes, Pezizomycetes, Leotiomycetes, Eurotiomycetes, Ascomycota_cls_Incertae_sedis, and the rest (others).

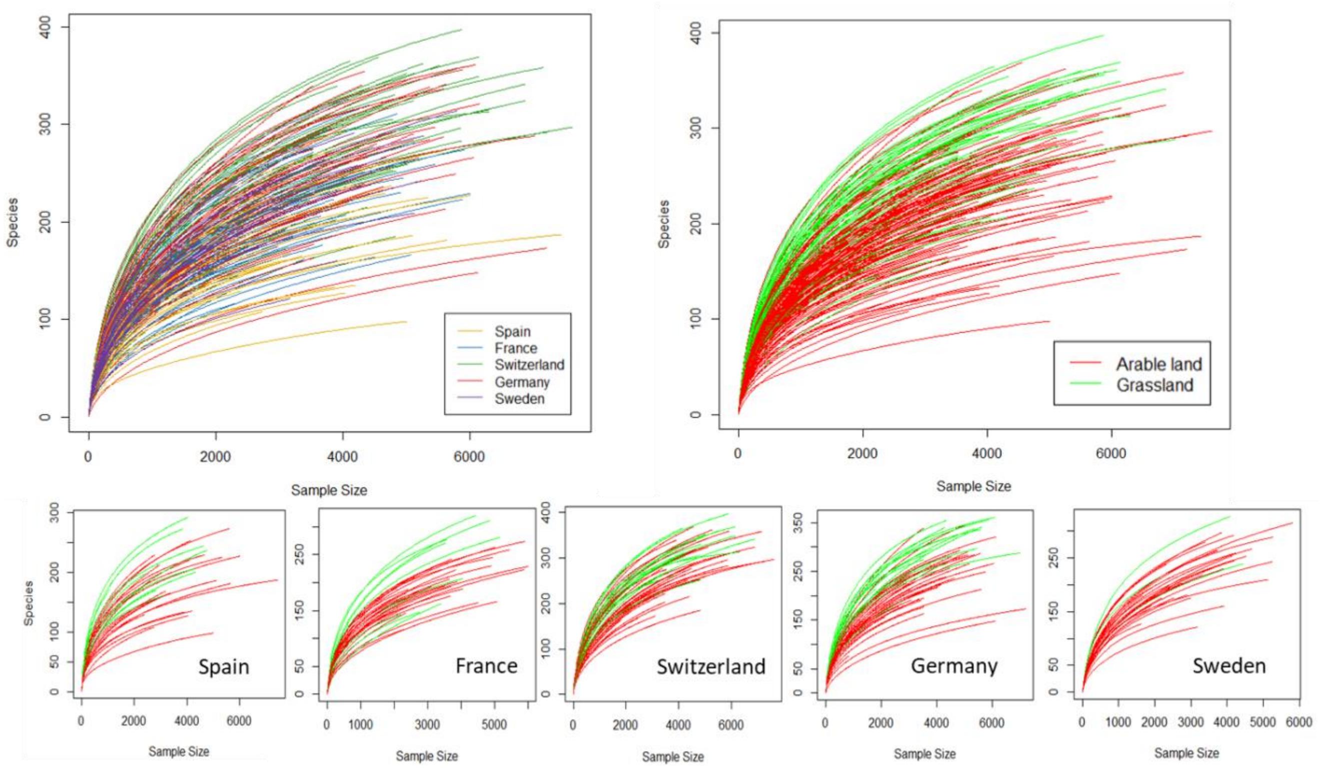

Figure S2. Rarefaction curves showing the number of fungal OTUs observed against the number of sequences sampled. **Top Panel-** overall rarefaction curve colored by countries (**left**) and land use types (**right**). **Bottom Panel-** Country-wise rarefaction curves colored by land use types.

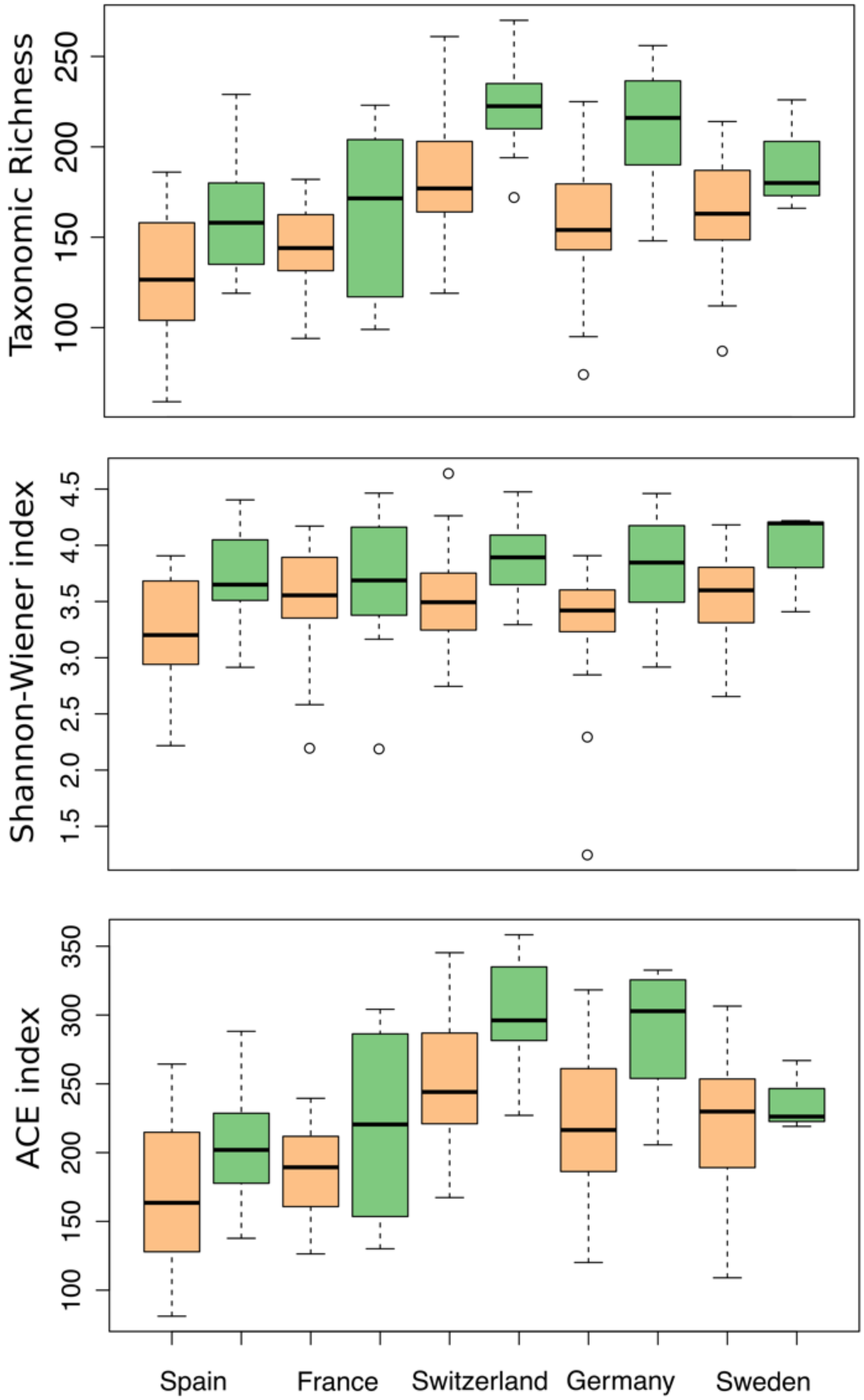

Figure S3. Alpha diversity indices of soil fungi in arable lands and grasslands of five countries across Europe.

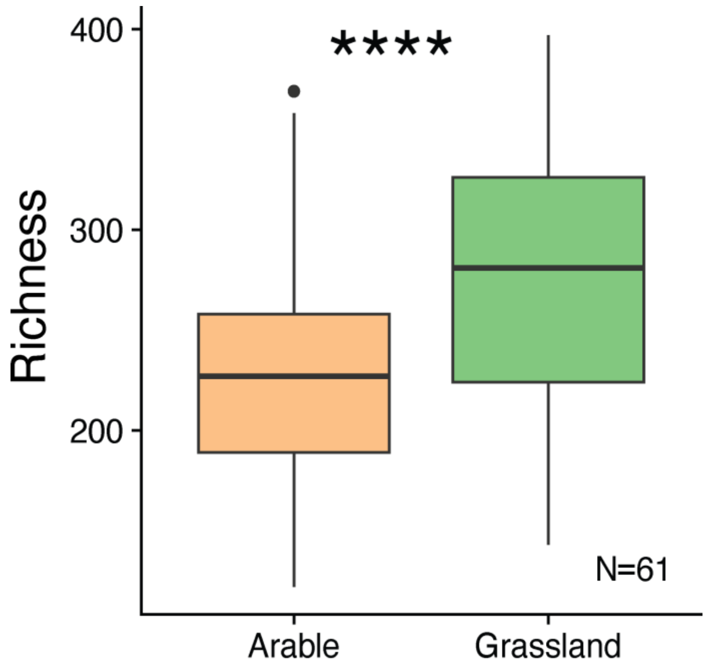

Figure S4. Fungal richness calculated for 61 samples in arable lands (randomly selected) and grasslands.

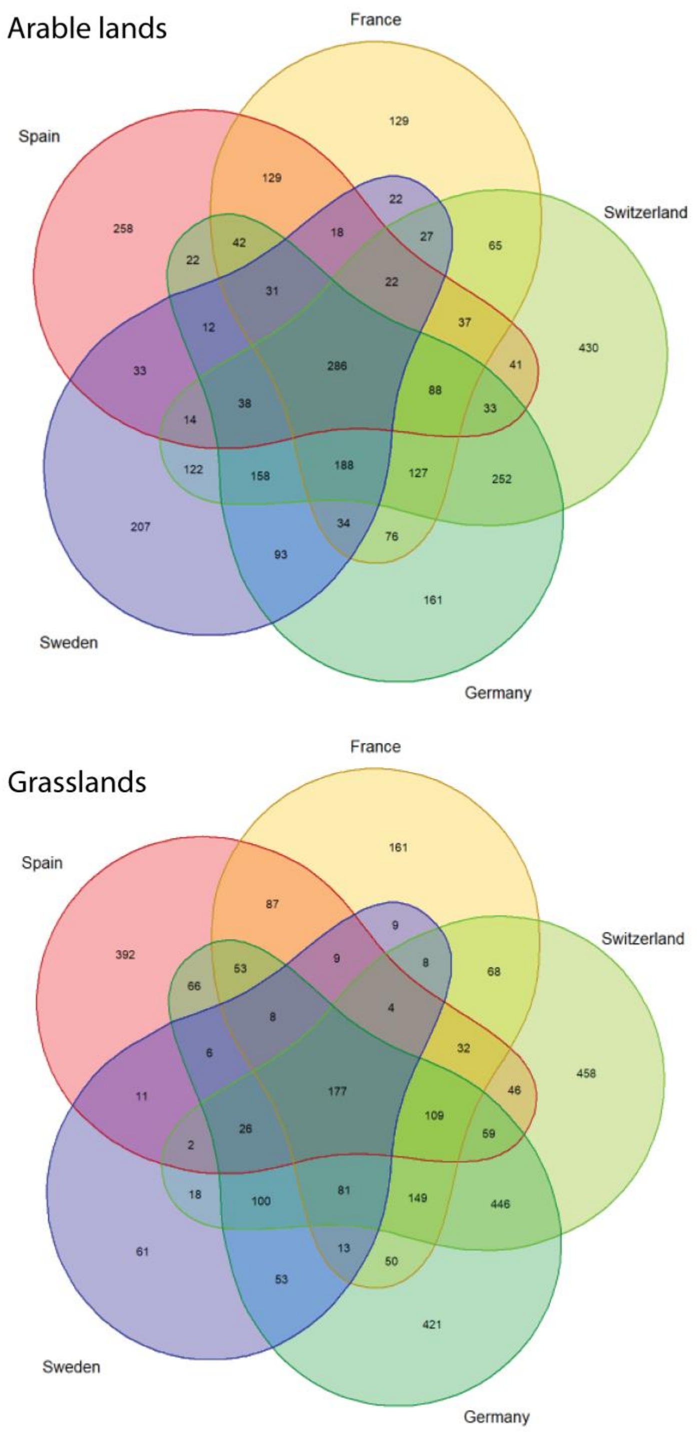

Figure S5: Venn diagrams showing the number of OTUs unique and common between five European countries.

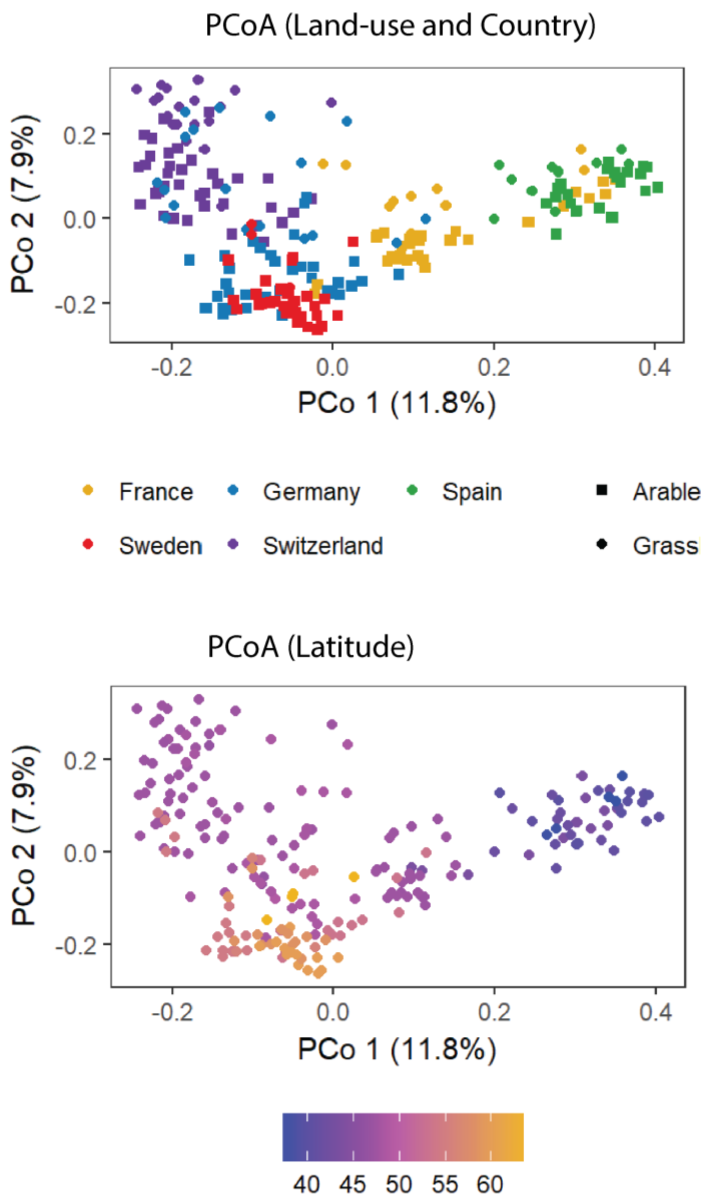

Figure S6. Principal coordinate analysis (PCoA) using Bray-Curtis similarity matrix soil fungal communities in arable lands and grasslands of five countries across Europe. **Upper Panel** shows fungal community structure across land-use and countries while **Lower Panel** shows patterns across latitudinal gradient.

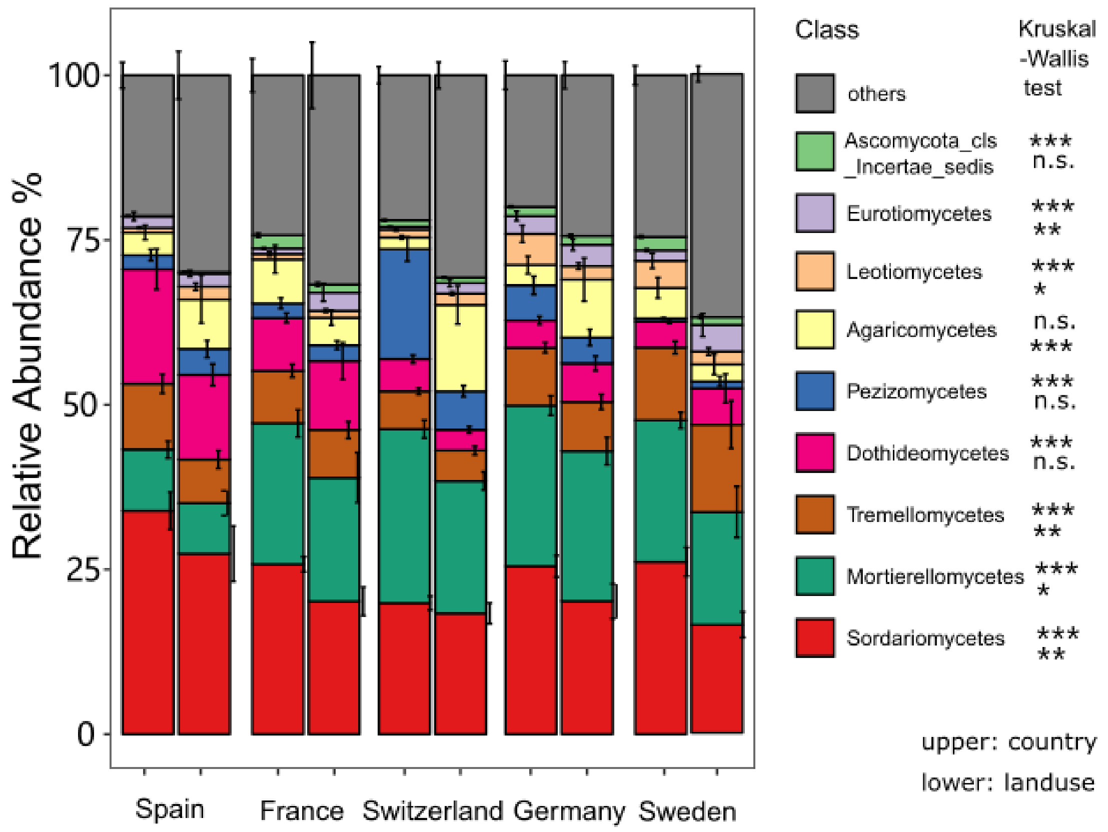

Figure S7. Relative abundance of soil fungal classes in five countries and two land use types. From left to right are countries from south to north. For each country, the left bar shows composition in arable lands and the right bar shows composition in grasslands. Kruskal-Wallis rank sum test was applied to test the significance of effects of country and land use type on the abundance of fungal classes. Statistical significance: * p value < 0.05; ** p value <0.01; *** p value <0.001; n.s. not significant.

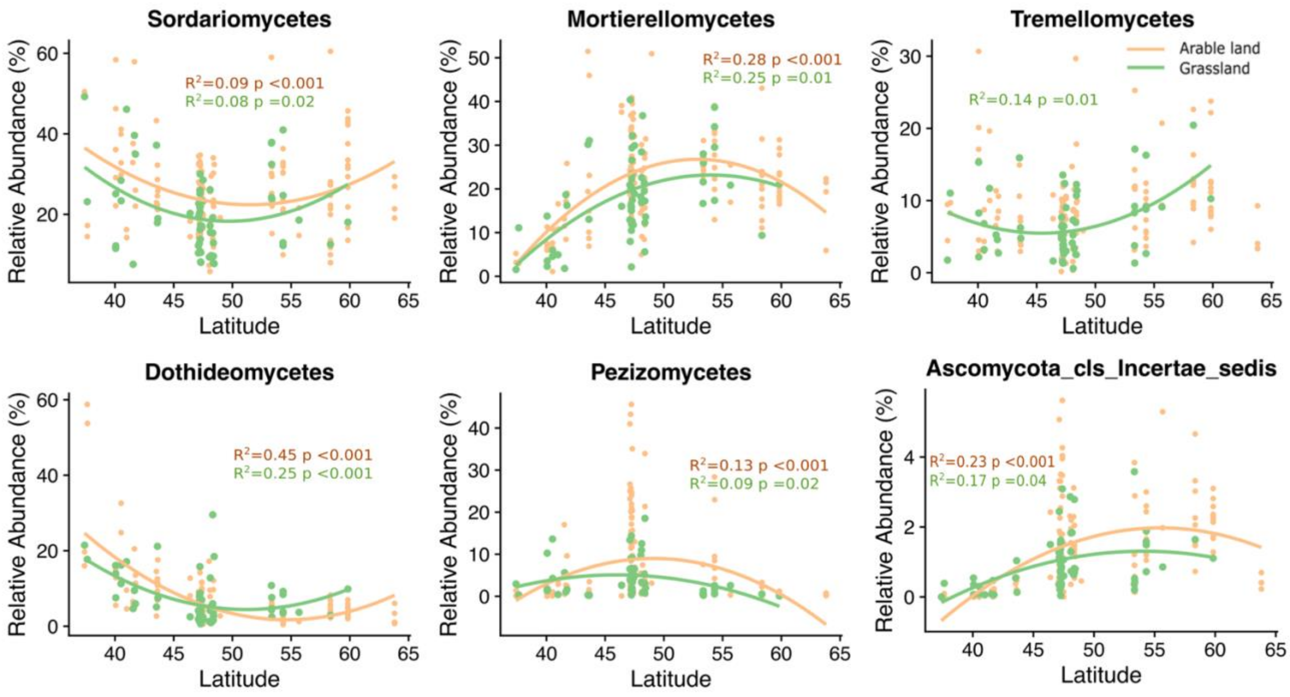

Figure S8. Latitudinal distribution of the six abundant fungal classes in arable lands and grasslands. Second- order polynomial models were fitted and plotted when fits were significant ($p<0.05$).

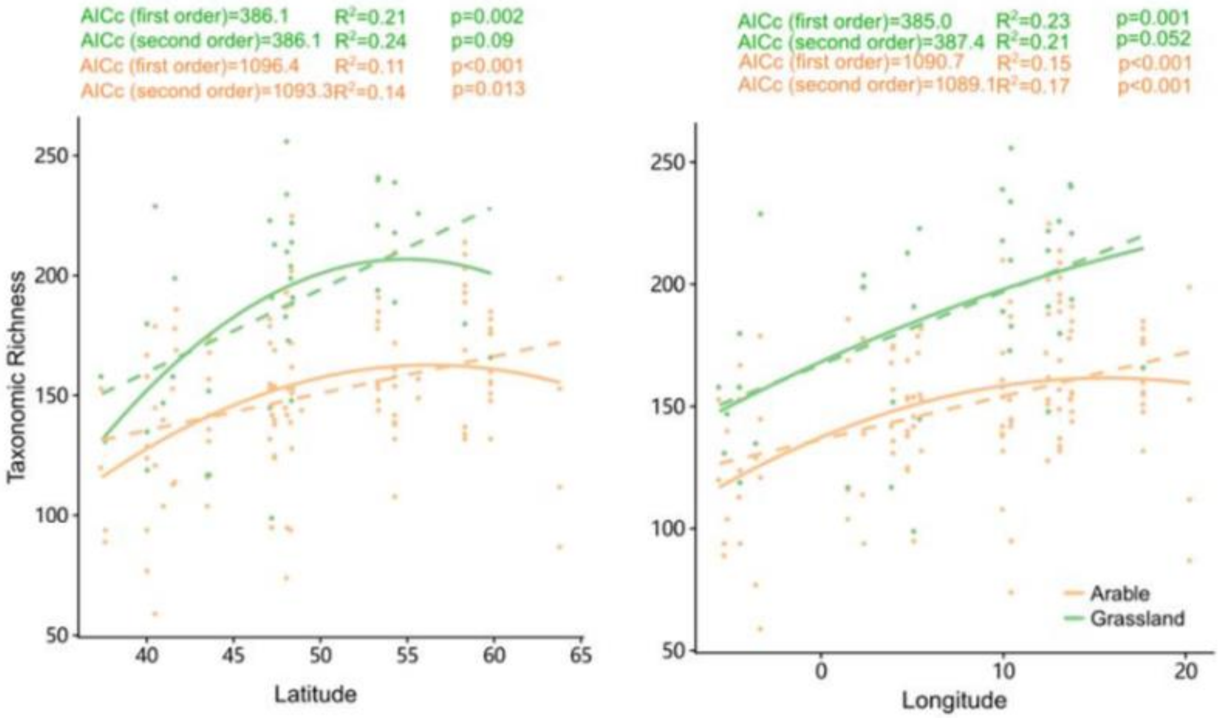

Figure S9. Latitudinal and longitudinal gradient of fungi richness without the data points from Switzerland.

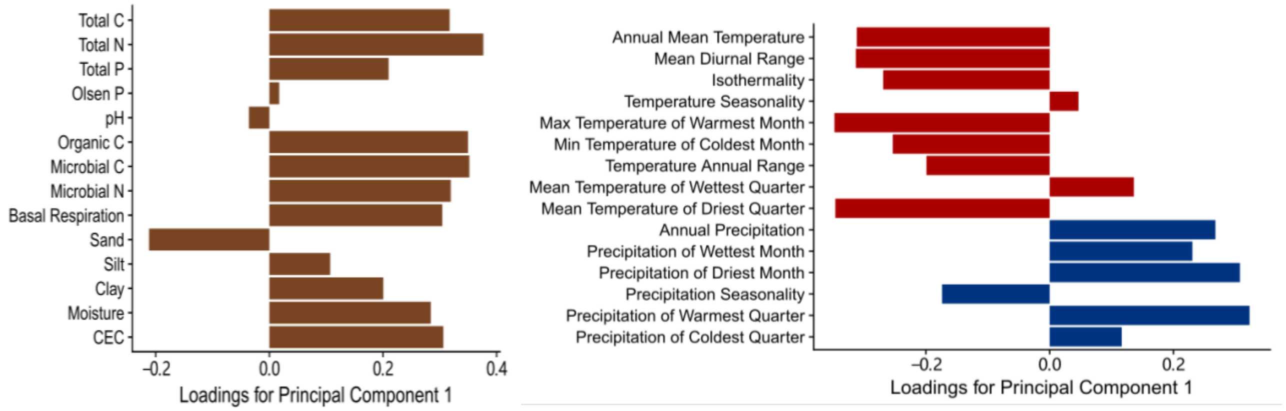

Figure S10. Loadings of principal component 1 of soil properties (**Left Panel**) and bioclimatic variables **(Right panel).**

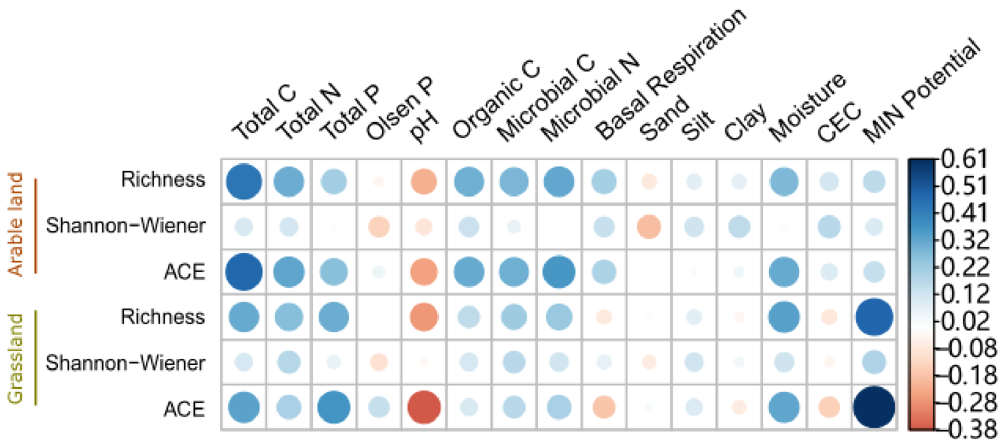

Figure S11. Correlogram showing Spearman correlations among soil properties and diversity indices. Circle size represents the strength of correlation.

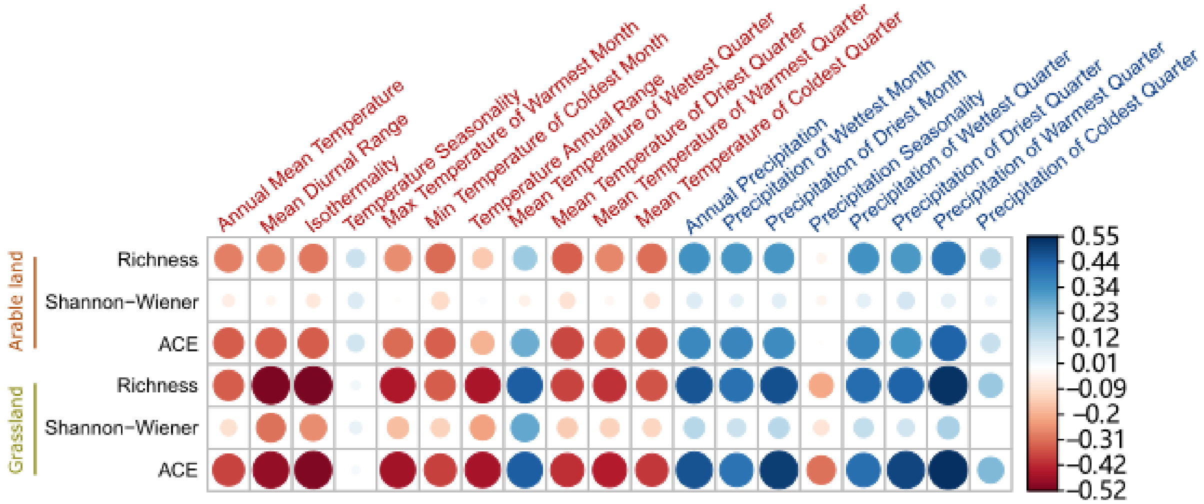

Figure S12. Correlogram showing Spearman correlations among bioclimatic variables and diversity indices. Circle size represents the strength of correlation.

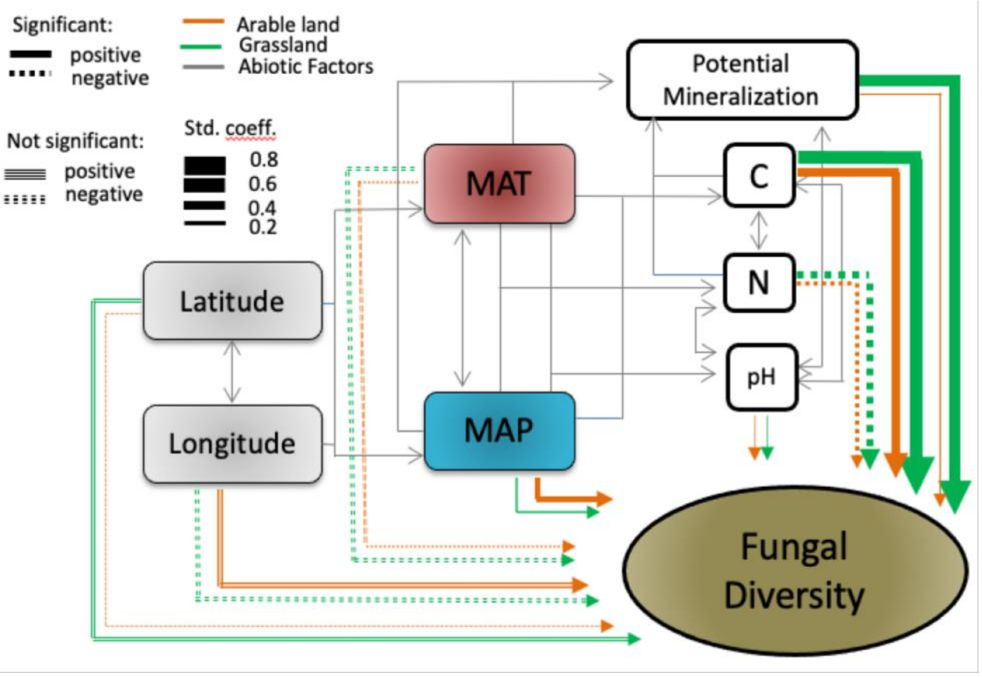

Figure S13. Structural equation modelling showing the drivers of fungal richness. Modelling was performed on all soil, geographical (latitude and longitude), and climatic (MAT: mean annual temperature and MAP: mean annual precipitation) variables. For soil properties, carbon and nitrogen contents, pH and potential mineralization displayed significant associations and thus included in the final model. Lines indicate significant paths, with increasing line thickness representing higher standardized coefficients. Single-headed arrows indicate direct effects while double-headed arrows indicate unresolved relationships. Positive relationships are shown with full lines and negative relationships with dotted lines.

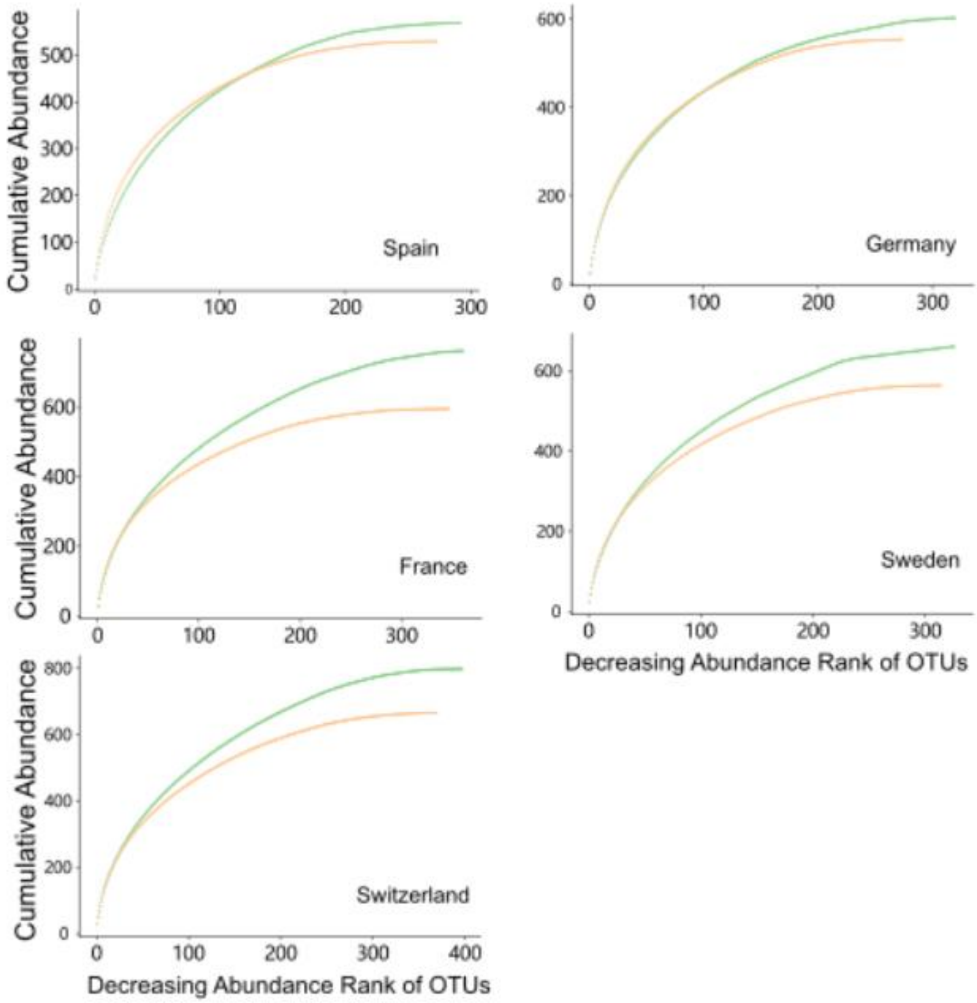

Figure S14. Cumulative abundance of OTUs in five European countries. Abundance of an OTU is calculated as the square-root transformed number of sequencing reads per sample. Cumulative abundance curves were created by averaging and summing up the ordered abundances.

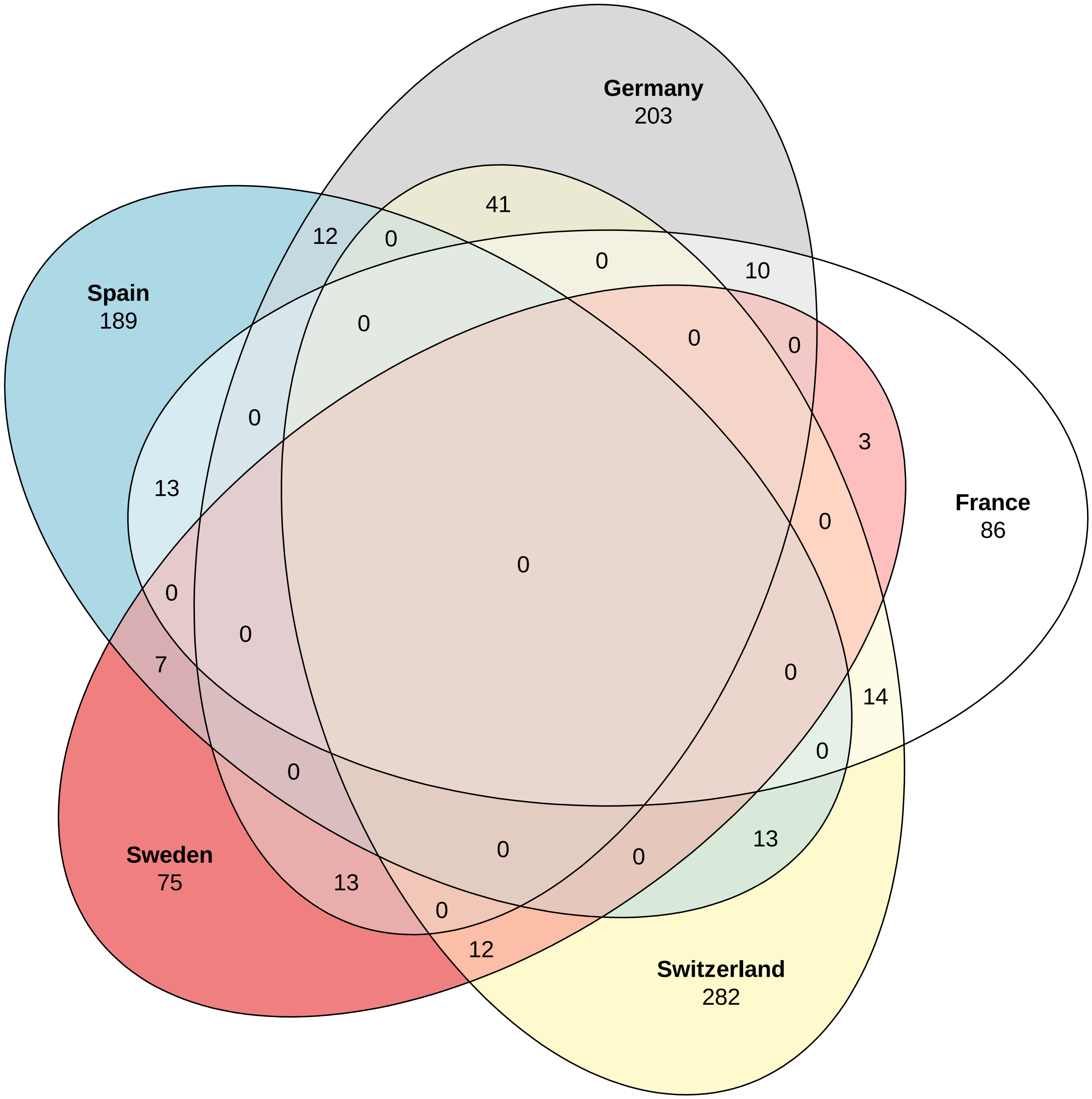

Figure S15. Venn diagram showing the number of unique and shared rare fungi.

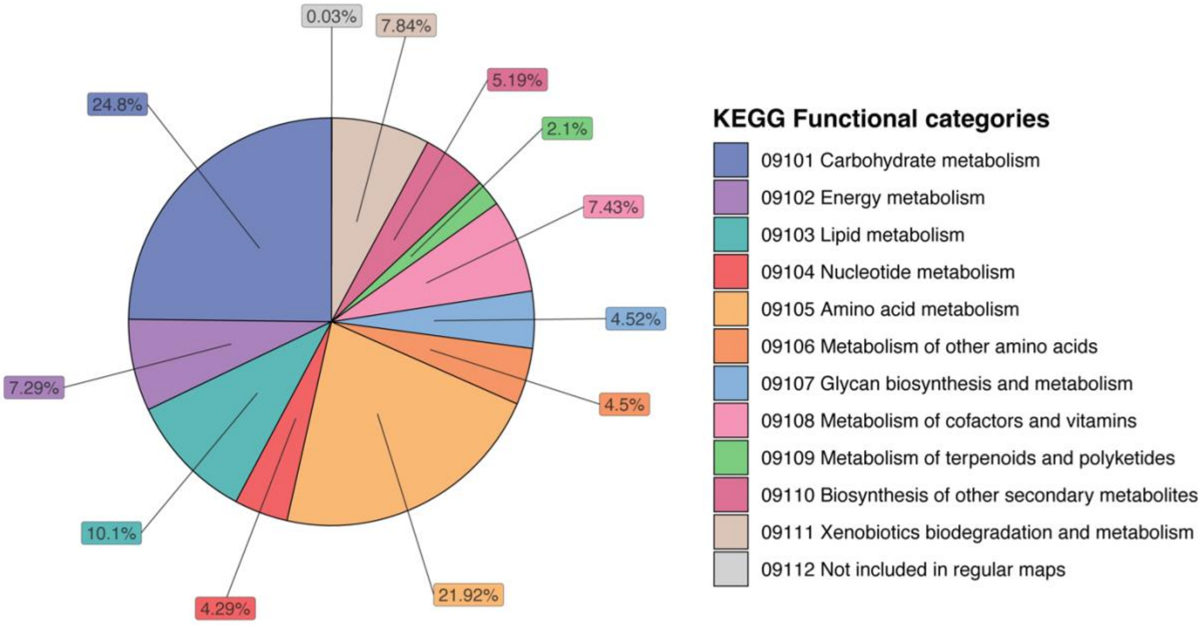

Figure S16. Molecular functions of rare fungi as shown by KEGG functional categories obtained through the FunFun pipeline, which is a functional annotator that evaluates the gene content of individual fungus from ITS sequencing data.

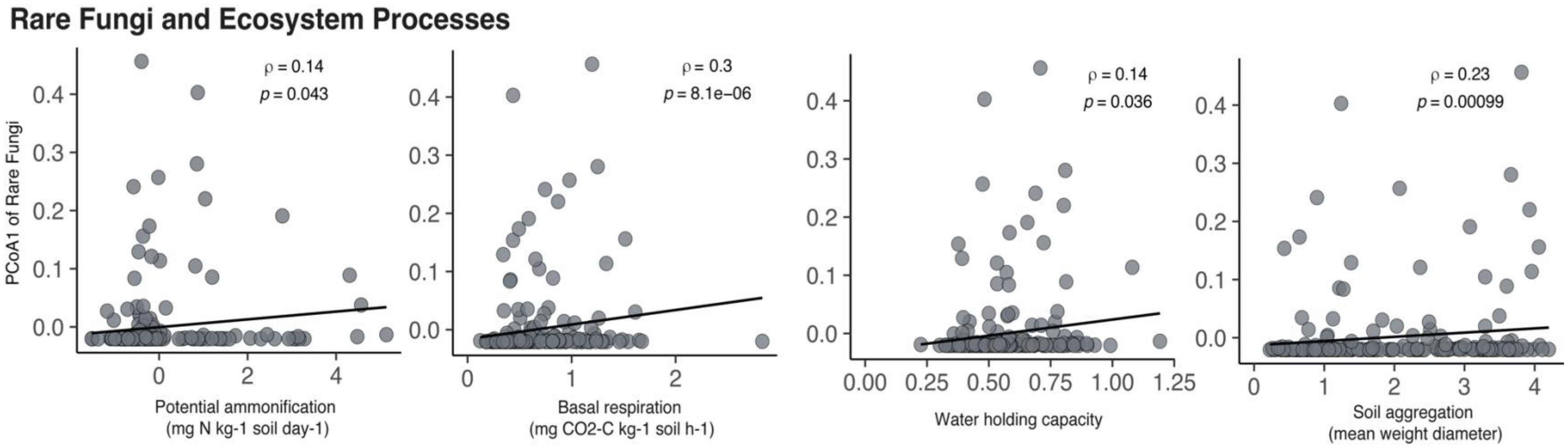

Figure S17. Relationships between rare soil fungi and important ecosystem processes across both land-use types. Rare fungal diversity is represented by the first Principal Coordinate calculated with a Bray-Curtis dissimilarity.

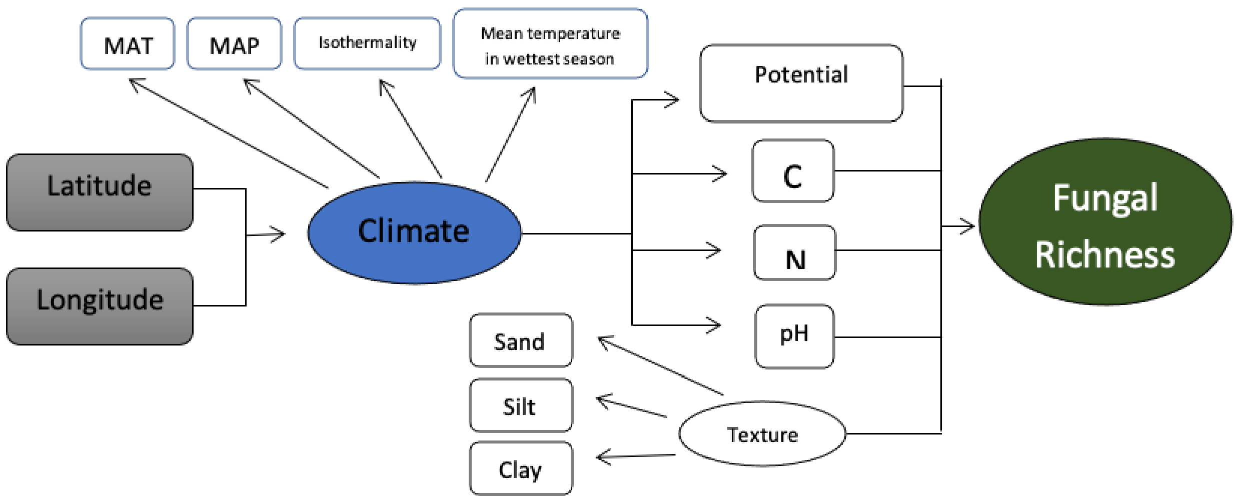

Figure S18. Initial structural equation model tested sequentially. We assumed that geographical location (latitude and longitude) influenced climatic factors, subsequently influencing soil properties, which thereby affected the soil fungal diversity. Latent variables are shown in ovals while observed variables are in rectangles.

Supplementary References

1. Gardes, M. & Bruns, T. D. ITS primers with enhanced specificity for basidiomycetes-application to the identification of mycorrhizae and ruts. *Mol. Ecol.* **2**, 113–118 (1993).
2. Lindahl, B. D. *et al.* Fungal community analysis by high-throughput sequencing of amplified markers--a user's guide. *New Phytol.* **199**, 288–99 (2013).
3. Schlaeppi, K. *et al.* High-resolution community profiling of arbuscular mycorrhizal fungi. *New Phytol.* **212**, 780–791 (2016).
4. Schmieder, R. & Edwards, R. Quality control and preprocessing of metagenomic datasets. *Bioinformatics* **27**, 863–864 (2011).
5. Edgar, R. C. Search and clustering orders of magnitude faster than BLAST. *Bioinformatics* **26**, 2460–2461 (2010).
6. Kõljalg, U. *et al.* UNITE: a database providing web-based methods for the molecular identification of ectomycorrhizal fungi. *New Phytol.* **166**, 1063–8 (2005).
7. Bengtsson-Palme, J. *et al.* Improved software detection and extraction of ITS1 and ITS2 from ribosomal ITS sequences of fungi and other eukaryotes for analysis of environmental sequencing data. *Methods Ecol. Evol.* **4**, 914–919 (2013).
8. Fick, S. E. & Hijmans, R. J. WorldClim 2: new 1-km spatial resolution climate surfaces for global land areas. *Int. J. Climatol.* **37**, 4302–4315 (2017).
9. Talbot, J. M. *et al.* Endemism and functional convergence across the North American soil mycobiome. *Proc. Natl. Acad. Sci.* **111**, 6341–6346 (2014).
