# Supplementary material for: Biotic homogenization, lower soil fungal diversity and fewer rare taxa in arable soils across Europe
Source: Nat Commun. 2024 Jan 6;15:327. doi: 10.1038/s41467-023-44073-6 (PMC10771452; doi:10.1038/s41467-023-44073-6)
Supplement: Supplementary file 2 — Reporting Summary [file 41467_2023_44073_MOESM2_ESM.pdf]

nature portfolio

Corresponding author(s): Samiran Banerjee, Marcel van der Heijden

Last updated by author(s): November 21, 2023

Reporting Summary

Nature Portfolio wishes to improve the reproducibility of the work that we publish. This form provides structure for consistency and transparency in reporting. For further information on Nature Portfolio policies, see our Editorial Policies and the Editorial Policy Checklist.

Statistics

For all statistical analyses, confirm that the following items are present in the figure legend, table legend, main text, or Methods section.

n/a	Confirmed	
☐	☒	The exact sample size (*n*) for each experimental group/condition, given as a discrete number and unit of measurement
☐	☒	A statement on whether measurements were taken from distinct samples or whether the same sample was measured repeatedly
☐	☒	The statistical test(s) used AND whether they are one- or two-sided<br>*Only common tests should be described solely by name; describe more complex techniques in the Methods section.*
☐	☒	A description of all covariates tested
☐	☒	A description of any assumptions or corrections, such as tests of normality and adjustment for multiple comparisons
☐	☒	A full description of the statistical parameters including central tendency (e.g. means) or other basic estimates (e.g. regression coefficient) AND variation (e.g. standard deviation) or associated estimates of uncertainty (e.g. confidence intervals)
☐	☒	For null hypothesis testing, the test statistic (e.g. *F*, *t*, *r*) with confidence intervals, effect sizes, degrees of freedom and *P* value noted<br>*Give P values as exact values whenever suitable.*
☒	☐	For Bayesian analysis, information on the choice of priors and Markov chain Monte Carlo settings
☒	☐	For hierarchical and complex designs, identification of the appropriate level for tests and full reporting of outcomes
☐	☒	Estimates of effect sizes (e.g. Cohen's *d*, Pearson's *r*), indicating how they were calculated

Our web collection on statistics for biologists contains articles on many of the points above.

Software and code

Policy information about availability of computer code

Data collection	All statistical analyses were conducted using packages in R (v.3.4.3).
Data analysis	All data and scripts are available on GitHub (https://github.com/sambanerjee2022/Agricultural-intensification-and-fungal-rarity.git. Sequences generated in this study are available through Sequence Read Archive (SRA) under BioProject accession number PRJNA1043689.

For manuscripts utilizing custom algorithms or software that are central to the research but not yet described in published literature, software must be made available to editors and reviewers. We strongly encourage code deposition in a community repository (e.g. GitHub). See the Nature Portfolio guidelines for submitting code & software for further information.

Data

Policy information about availability of data

All manuscripts must include a data availability statement. This statement should provide the following information, where applicable:

- Accession codes, unique identifiers, or web links for publicly available datasets
- A description of any restrictions on data availability
- For clinical datasets or third party data, please ensure that the statement adheres to our policy

All data and scripts are available on GitHub (https://github.com/sambanerjee2022/Agricultural-intensification-and-fungal-rarity.git. Sequences generated in this study are available through Sequence Read Archive (SRA) under BioProject accession number PRJNA1043689.

March 2021

Human research participants

Policy information about studies involving human research participants and Sex and Gender in Research.

Reporting on sex and gender	NA
Population characteristics	NA
Recruitment	NA
Ethics oversight	NA

Note that full information on the approval of the study protocol must also be provided in the manuscript.

Field-specific reporting

Please select the one below that is the best fit for your research. If you are not sure, read the appropriate sections before making your selection.

☐ Life sciences ☐ Behavioural & social sciences ☒ Ecological, evolutionary & environmental sciences

For a reference copy of the document with all sections, see nature.com/documents/nr-reporting-summary-flat.pdf

Ecological, evolutionary & environmental sciences study design

All studies must disclose on these points even when the disclosure is negative.

Study description	Soil fungi are a key constituent of global biodiversity and play a pivotal role in agroecosystems. How agricultural intensification affects soil microbial biogeography is poorly understood. This study was a part of the Digging Deeper Project conducted across five European countries Sweden, Germany, Switzerland, France, and Spain. A total of 217 agricultural fields were chosen, including 156 arable sites and 61 extensively managed grasslands.
Research sample	We assessed soil fungal communities by collecting soil samples from topsoil (20 cm depth).
Sampling strategy	In Spring 2017, we sampled soils form 156 croplands and 61 extensively managed grasslands sites across five European countries. A total of 217 agricultural fields were chosen, including 156 arable sites and 61 extensively managed grasslands28,52. A majority (78%) of the arable sites were planted with wheat (Triticum aestivum) (n= 121) with the other cereal crops such as barley, Hordeum vulgare (n=26); oat, Avena sativa (n=6); rye, Secale cereale (n=1); or triticale, Triticosecale sp. (n=1) selected when wheat was unavailable. When possible, we paired agricultural fields with non-arable lands by sampling nearby extensively managed grasslands and marginal lands with permanent, predominantly herbaceous plant cover.
Data collection	At each site, eight soil cores were obtained in a circular pattern within a 10 m radius using a 5 cm diameter step-probe and to a depth of 20 cm. Team members labeled the samples using a permanent marker. Soil samples were kept on ice until their transfer to the laboratory. Three of the cores were kept intact and used to measure bulk density and soil aggregation. The remaining soil cores were homogenized and sieved to 2 mm. Soil sub-samples were air-dried for further processing for soil physical and chemical properties, stored at 4°C for soil properties such as microbial biomass, and frozen at -18°C for DNA extraction, mineral nitrogen content and potential N cycling rates.
Timing and spatial scale	All samples were collected during the crop flowering period in each site (between May and June 2017). These sampling times were chosen based on the crop growth and weather patterns. The entire sampling was completed within three weeks.
Data exclusions	No data excluded
Reproducibility	All laboratory analyses were performed with two analytical replicates to ensure that data was reproducible. Different statistical tests were run at least three times to check consistency of findings.
Randomization	During all laboratory analyses, the samples were measured in a completely randomized fashion in order to avoid bias. This was done by re-naming each sample as a number, so all information regarding individual samples was not known during analysis.
Blinding	Each sample was re-named by randomly assigning a number. This allowed all laboratory analyses to be conducted in a completely randomized fashion, without knowledge of which sample was being analyzed.

Did the study involve field work? ☒ Yes ☐ No

Field work, collection and transport

Field conditions	During the spring of 2017, we sampled 217 sites across a North-South gradient of Europe. All background data are available at- figshare.com/articles/dataset/Garland_et_al_Nat_Food_dataset_figshare_xlsx/10067999
Location	Five countries: Spain, France, Switzerland, Germany, and Sweden. Site locations (regions, latitude, longitude etc.) are available at- figshare.com/articles/dataset/Garland_et_al_Nat_Food_dataset_figshare_xlsx/10067999
Access & import/export	Since all soils are located within Europe, we did not require special permits to transfer the soil samples between research groups. Sites were accessed using vehicles. Samples were always transported on ice to ensure integrity of samples.
Disturbance	There was minimal disturbance to the farmers' fields during this study, in which we only took 8 soil cores from each site in a 10-m radius. At each site, team members walked between rows and avoided trampling plants. Additionally, all farmers were financially compensated at an amount appropriate for each country for allowing us to sample their fields.

Reporting for specific materials, systems and methods

We require information from authors about some types of materials, experimental systems and methods used in many studies. Here, indicate whether each material, system or method listed is relevant to your study. If you are not sure if a list item applies to your research, read the appropriate section before selecting a response.

Materials & experimental systems

n/a	Involved in the study
☒	☐ Antibodies
☒	☐ Eukaryotic cell lines
☒	☐ Palaeontology and archaeology
☒	☐ Animals and other organisms
☒	☐ Clinical data
☒	☐ Dual use research of concern

Methods

n/a	Involved in the study
☒	☐ ChIP-seq
☒	☐ Flow cytometry
☒	☐ MRI-based neuroimaging
